# *Coccinia intermedia* – a new Cucurbitaceae species from West Africa

**DOI:** 10.3897/phytokeys.7.2032

**Published:** 2011-11-29

**Authors:** Norbert Holstein, Susanne S. Renner

**Affiliations:** 1 Systematic Botany and Mycology, Menzinger Str. 67, 80638 Munich, Germany

**Keywords:** Benin, Ivory Coast, Ghana, leaky dioecy, molecular phylogenetics, species monophyly, Togo

## Abstract

Nuclear and plastid sequences from two individuals of a suspected new species of *Coccinia* from West Africa were added to an available molecular phylogeny for the remaining 27 species of the genus. Phylogenetic analyses of these data indicate the new species' monophyletic status and closest relatives. Based on four fertile collections, we here describe and illustrate *Coccinia intermedia* Holstein. We also provide a key to the *Coccinia* species of West Africa and map their distributions.

## Introduction

The genus *Coccinia* Wight et Arn. so far consisted of 27 species distributed mainly in Sub-Saharan Africa, with centers of diversity in East Africa and southern Africa (Holstein, ongoing monograph). Only four species were known from West Africa, including *Coccinia longicarpa* Jongkind, *Coccinia keayana* R. Fern., and *Coccinia barteri* (Hook. f.) Keay, which apparently evolved during Pliocene-Pleistocene climatic oscillations (Holstein and Renner 2011). The fourth species, *Coccinia grandis* (L.) Voigt, is much more widespread, occurring not only in Africa but also in South and South East Asia, and being naturalized on several Pacific islands, Australia, and in the Neotropics. During a study of the evolution and biogeography of the genus (Holstein and Renner 2011), we came across a male specimen from the northeastern Ivory Coast that in its plastid sequences differed sufficiently from all other sequenced material for us to suspect it might represent a new species. We therefore provisionally labeled it *Coccinia* sp. nov. We have since found three more specimens of the new species, all of them with fruits, and two with flowers, and based on their morphology as well as additional nuclear and plastid sequences, we here describe the new species *Coccinia intermedia*.

## Methods

We produced new sequences of the plastid *rpl*20–*rps*12 intergenic spacer (JN653687), *trn*S^GCU^–*trn*G^UCC^ intergenic spacer (JN653686) and the nuclear *LEAFY*-like second intron (JN653688) from the female specimen A. Akoègninou et al. 2625 (WAG0278370) of the new species, following standard procedures (Holstein and Renner 2011). We added the new sequences, named “*Coccinia intermedia* 2”, to our published matrices and carried out maximum likelihood tree searches, using the approaches described in Holstein and Renner (2011).

## Results

### Phylogenetic placement

**Figure 1.**
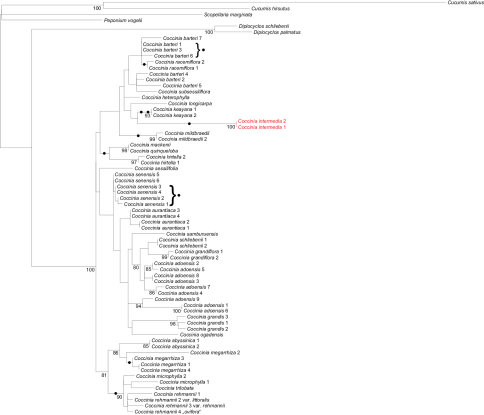
Maximum likelihood phylogeny for *Coccinia* based on plastid DNA sequences analyzed under GTR+Γ model of substitution. The tree is based on 4,551 nucleotides (140 parsimony-informative sites) from the *trn*S^GCU^–*trn*G^UCC^ intergenic spacer (IS), the *rpl*20–*rps*12 IS, the *ndh*F–*rpl*32 IS, *trn*L^UAA^ intron, *trn*L^UAA^–*trn*F^GAA^ IS, and the *mat*K gene (expanded matrix from Holstein and Renner 2011). Numbers below branches represent bootstrap support ≥ 80% from 1000 replicates. Dots on branches and behind brackets refer to uniquely shared insertions or deletions. Species names follow Holstein and Renner (2011) except for the new species *Coccinia intermedia* 1, earlier called *Coccinia* sp. nov.

**Figure 2.**
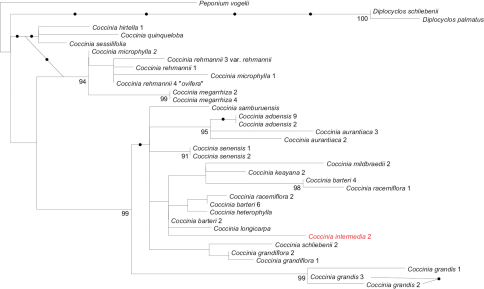
Maximum likelihood phylogeny for *Coccinia* based on nuclear DNA sequences from the *LEAFY*-like 2^nd^ intron analyzed under the GTR+Γ model of substitution. The tree is based on 505 nucleotides (56 parsimony-informative sites). Numbers below branches represent bootstrap support ≥ 80% from 100 replicates. Dots on branches and behind brackets refer to uniquely shared insertions or deletions. Species names follow Holstein and Renner (2011) except for the new species *Coccinia intermedia*.

The two *Coccinia intermedia* accessions in the plastid tree form a clade (Fig. 1) within the *barteri* clade. In the nuclear *LEAFY* phylogeny, *Coccinia intermedia* groups with *Coccinia barteri*, *Coccinia heterophylla* (Hook.f.) Holstein, *Coccinia keayana*, *Coccinia longicarpa*, *Coccinia mildbraedii* Gilg, and *Coccinia racemiflora* Keraudren (Fig. 2), albeit without bootstrap support.

### Morphological description

#### Coccinia intermedia sp. nov.

urn:lsid:ipni.org:names:77115897-1

http://species-id.net/wiki/Coccinia_intermedia

##### Latin.

A *Coccinia longicarpa* differt calycis dentibus angustis, corolla campanulata et fructu elliptico ad oblongo. A *Coccinia keayana* et *Coccinia grandis* differt calycis dentibus ad corollam adpressis vel apicem versus leviter recurvatis et lamina foliorum subtus glandibus fuscis provisa. A *Coccinia barteri* differt floribus femineis 1–3 fasciculatis non racemosis, corolla campanulata.

##### Type.

 BENIN. Atakora: Natitingou, Kouaténa (Perma), 10°12.00'N; 1°30.18'E, river bed, female, fl, fr, 3 Oct 2000, A.Akoègninou et al. 3625 (Holotype: WAG0278370!; isotype: WAG0278369!).

##### Description.

**Figure 3.**
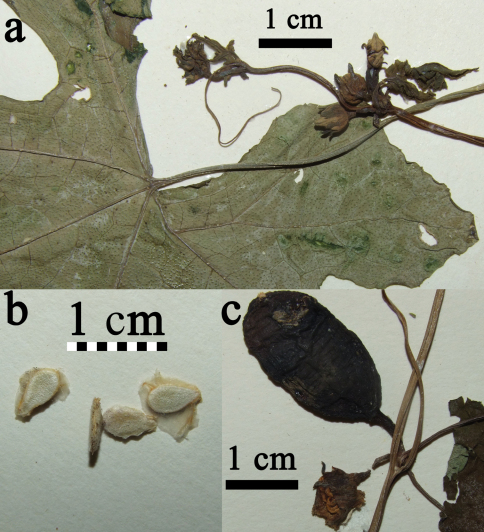
**a** *Coccinia intermedia* leaf basis and node with flowers **b** seeds from late, but immature fruit  **c** node with young fruit and male flower bud with sinuate anthers; all from J.B.Hall & J.M.Lock GC46016 (K).

**Figure 4.**
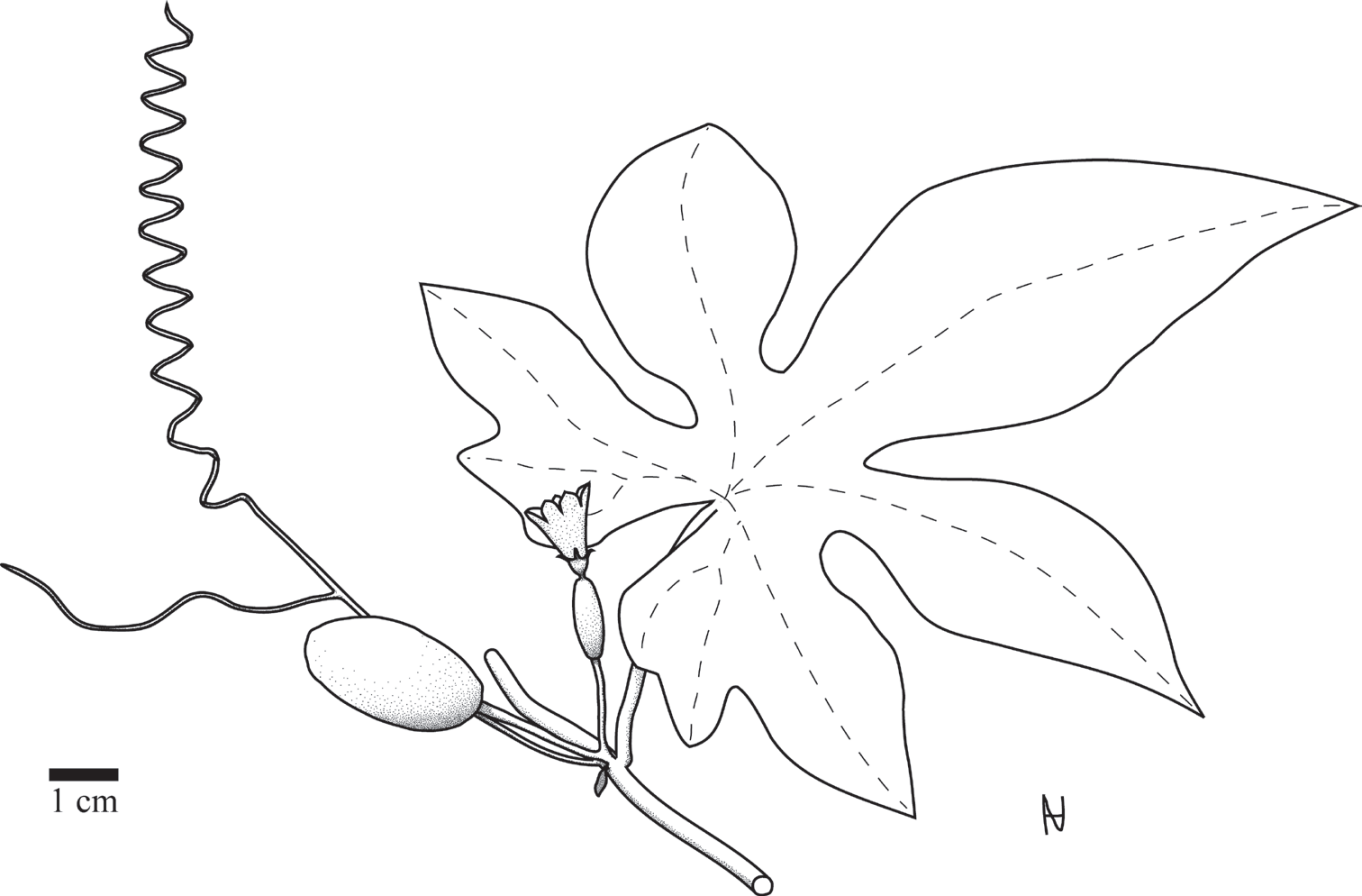
Habitus of *Coccinia intermedia* as reconstructed from J.B.Hall & J.M.Lock GC46016 (K).

**Figure 5.**
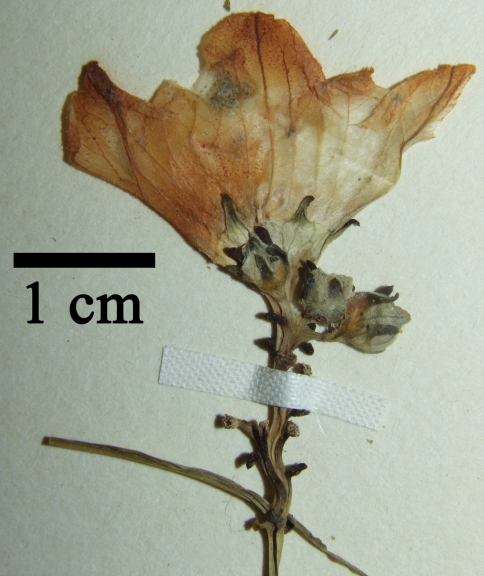
Male inflorescence of *Coccinia intermedia* from C.Geerling & J.Bokdam 662 (WAG).

Perennial, diclinous climber. Shoot length unknown, but likely several meters. Shoots lignify with whitish bark and up to 1 cm diam. Fresh shoots green, glabrous, older shoots with clear to white pustules. Petioles 2.8–10.8 cm, glabrous, when older with clear to white pustules (Fig. 3a). Leaves 6–15 × 7–18 cm, shallowly to profoundly 5-lobate, more or less auriculate (Fig. 4). Upper lamina glabrous with clear to whitish pustules. Lower lamina paler than upper lamina, glabrous, often with small dark glands near the leaf base (Fig. 3a). Tendrils simple or bifid. Probracts up to 2.5 mm long, glabrous, apex rounded (Fig. 3a). Male flowers in few-flowered racemes (Fig. 5), likely sometimes accompanied by a single flower. Common peduncle up to 1 cm, pedicels in racemose flowers 2–4 mm, glabrous. Bracts up to 1.5 mm long, round to obovoid. Receptacle pale green, glabrous. Calyx teeth 1.5 mm long, lineal to narrow triangulate, erect with slightly recurved tips (Figs 3–5). Corolla campanulate, 1.6 cm long, pale reddish-yellow to yellow, lobes 0.7 cm long (Fig. 5). Anthers sinuate, in a globose head (Fig. 3c). Pollen unknown. Female flowers 1–3 clustered (strongly reduced raceme; Fig. 4). Pedicels 0.6–1.2 cm, glabrous. Perianth like in males. Ovary fusiform, glabrous. Stigma and staminodes unknown. Fruit 4.5 × 2.5 cm, elliptical to oblong, smooth. Unripe green with pale green longitudinal mottling. Ripe orange?, more likely becoming red via orange ripening stage. Fruit with waxy cover. Size of mature seeds unknown (≥ 5.5 × 3.5 × 1.3 mm), symmetrical (to slightly asymmetrical), face flat (Fig. 3b).

##### Distribution.

**Figure 6.**
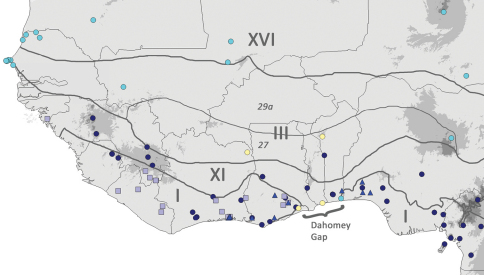
Map of West African *Coccinia* species. Pale yellow circles = *Coccinia intermedia*, cyan circles = *Coccinia grandis*, dark blue circles = *Coccinia barteri*, pale blue squares = *Coccinia keayana*, bright blue triangles = *Coccinia longicarpa*. Thick dark grey lines are phytochoria drawn after White (1983), I = Guineo-Congolian regional center of endemism, III = Sudanian regional center of endemism, XI = Guinea-Congolia/Sudania transition zone, XVI = Sahel regional transition zone. Thin dark grey lines (after White (1983)) differentiate between White’s vegetation types of zone III: 27 = Sudanian woodland with abundant *Isoberlinia*; 29a = undifferentiated Sudanian woodland. Location of *Coccinia intermedia* in Ivory Coast estimated (only the department is given on the herbarium sheet).

(Fig. 6). NE Ivory Coast, SE Ghana (likely also in the north), S Togo (likely also in the north), NW Benin. Based on the current collections, *Coccinia intermedia* is likely to occur in the Dahomey Gap region and the *Isoberlinia* woodlands of West Africa.

##### Ecology.

 Wooded grasslands (semi-humid savanna), woodlands, dry forests, and along rivers. Flowering specimens have been collected during May, August, and October, which in each site was during or shortly after the rainy season.

##### Etymology.

 The epithet refers to the species' status as the only *Coccinia* from West Africa that occurs in habitats intermediate between semi-arid and humid conditions. Morphologically, *Coccinia intermedia* combines characters also found in the other four West African species although not in this combination.

##### List of specimens examined.

 Benin: Atakora, Natitingou, Kouaténa (Perma), 10°12.00'N; 1°30.18'E, river bed, female, fl, fr, 3 Oct 2000, A.Akoègninou et al. 3625 (WAG 2 sheets). Ghana: Shai Hills Game Reserve, monoecious, fl, fr, 25 May 1976, J.B.Hall & J.M.Lock GC 46016 (K 4 sheets, MO). Ivory Coast: Bouna, male, fl, 10 Aug 1967, C.Geerling & J.Bokdam 662 (MO, WAG). Togo: between Lomé and Aného, female, fr, 25 Jun 1994, L.Aké Assi 18982 (MO).

### Key to West African Coccinia species

**Table:** 

1	Plant glabrous. Leaves with few large pale glands between main nerves of lower lamina. Nerves on lower lamina with or without white pustules. Leaf margin dentate, in mature plants often red to brown (black when dry). Tendrils always simple. Male and female flowers 1 solitary (rarely male flowers clustered or in short-peduncled racemes). Calyx teeth spreading to reflexed, tips red to brown. Corolla campanulate, white or buff. Fruit ovoid. Plant of semi-arid habitats.	*Coccinia grandis*
1'	Plant glabrous or with hairs, especially on adaxial petiole. Leaves with small blackish glands (often many) centered towards the leaf base or without glands on lower lamina. Tendrils simple or bifid. Male and female flowers in racemes or solitary. Corolla in yellowish tones, never white.	2
2	Plant glabrous. Leaves with small blackish glands centered towards the leaf base (Fig. 3). Nerves on lower lamina with or without white pustules. Leaf margin at maturity with colored teeth (color in living plants unknown, black when dry). Tendrils simple or bifid. Male flowers (Fig. 5) bracteate, in few-flowered racemes, female flowers 1–3 solitary/clustered (Figs 3 and 4). Calyx teeth erect with recurved tips (Figs 3–5). Corolla campanulate. Fruit ovoid to short cylindrical. Plant of wooded grasslands (tree savanna), woodlands, or dry forests.	*Coccinia intermedia*
2'	Plant glabrous or with hairs, esp. on adaxial petiole. Leaves with small blackish glands centered towards the leaf base or without glands. Nerves on lower leaf lamina without white pustules. Tendrils simple or bifid. Male flowers in few to many-flowered racemes, rarely accompanied by a solitary flower. Female flowers in few- to many-flowered racemes or solitary. Flowers bracteate or ebracteate. Corolla urn-, cup- to funnel-shaped. Plant of humid climates (rainforests, gallery forests, etc.)	3
3	Leaf margin with pale (when dry blackening) glandular teeth. Tendrils simple. Flowers without bracts, calyx teeth erect, > 1.5 mm at base. Fruits long cylindrical.	*Coccinia longicarpa*
3'	Leaf margin without conspicuously colored teeth. Tendrils simple or bifid. Flowers with or without bracts. Calyx teeth erect, spreading, or reflexed, but narrow (< 1.2 mm at base). Fruits ovoid.	4
4	Tendrils simple. Male flowers in lax racemes, female flowers solitary or in few-flowered racemes. Flowers without bracts. Calyx teeth in buds spreading, later reflexed.	*Coccinia keayana*
4'	Tendrils simple or bifid. Male flowers in dense few- to many-flowered racemes, with or without bracts. Female flowers in racemes, rarely solitary. Flowers with or without bracts. Calyx teeth variable.	*Coccinia barteri*

## Discussion

*Coccinia intermedia* is morphologically similar to the other West African species. From *Coccinia grandis*, it differs most readily in the glands on the lower lamina and in its calyx teeth (erect with recurved tips in *Coccinia intermedia* and spreading to reflexed in *Coccinia grandis*). From *Coccinia longicarpa*, it differs in its ovoid fruits (instead of long cylindrical fruits in *Coccinia longicarpa*). Additionally, *Coccinia longicarpa* has ebracteate racemes and much broader (> 1.5 mm at the base) erect calyx teeth, and an urn-shaped corolla. From *Coccinia keayana*, it differs in having bracteate male flowers in denser racemes, a campanulate corolla and calyx teeth that are adpressed to the corolla with recurved tips, instead of spreading (in buds) to reflexed calyx teeth. Secure distinction of *Coccinia intermedia* from *Coccinia barteri* requires fertile material with flowers (see the key above).

Ecologically, the new species is a member of White's (1983) Sudanian center of endemism and his Guinea-Congolia/Sudania regional transition zone (Fig. 6). The only species with a similar habitat as *Coccinia intermedia* is *Coccinia adoensis*, the most western known occurrence of which is Adamawa State (eastern Nigeria). Whether the species co-occur is not known. They could be distinguished by fruit shape (not beaked in *Coccinia intermedia*, beaked in *Coccinia adoensis*, although this character can vary in the latter).Additionally, *Coccinia adoensis* has inflorescence peduncles that are longer than 1 cm (in its male racemes) and petioles that are often hairy.

Two DNA characters, namely base pairs 310 and 323 in the *trn*S^GCU^–*trn*G^UCC^ intergenic spacer region, suggest the placement of *Coccinia intermedia* as sister to a clade that we have earlier referred to as the *Coccinia barteri* clade (Holstein and Renner 2011). If this placement is correct, then the *Coccinia* species occurring in the rain or mist forests of West and Central African are monophyletic and probably evolved *in situ*. One of the four collections, J.B.Hall & J.M.Lock GC 46016, bears male and female flowers/fruits on the same node (Fig. 3c). The male flowers are buds, and it is not clear, whether they are fertile. Kumar and Vishveshwaraiah (1952) report a “gynodioecious form” of *Coccinia grandis* in which the male flowers of the hermaphrodite (monoecious) plants are sterile. An occasional occurrence of bisexual plants in otherwise dioecious species, sometimes called “leaky dioecy” (Baker and Cox 1984), has also been observed in other Cucurbitaceae (Schaefer and Renner 2010).

However, true monoecy in *Coccinia intermedia* would be surprising as none of ca. 1,500 specimens of other *Coccinia* species studied is bisexual (Holstein, ongoing monograph).

## Supplementary Material

XML Treatment for Coccinia intermedia
